# LM11A-31, a modulator of p75 neurotrophin receptor, suppresses HIV-1 replication and inflammatory response in macrophages

**DOI:** 10.3389/ebm.2024.10123

**Published:** 2024-07-25

**Authors:** Golnoush Mirzahosseini, Namita Sinha, Lina Zhou, Sandip Godse, Sunitha Kodidela, Udai P. Singh, Tauheed Ishrat, Santosh Kumar

**Affiliations:** ^1^ Department of Pharmaceutical Sciences, College of Pharmacy, The University of Tennessee Health Science Center, Memphis, TN, United States; ^2^ Department of Anatomy and Neurobiology, College of Medicine, The University of Tennessee Health Science Center, Memphis, TN, United States

**Keywords:** LM11A-31, HIV-1, inflammation, macrophages, oxidative stress, cytotoxicity

## Abstract

Antiretroviral drugs have made significant progress in treating HIV-1 and improving the quality of HIV-1-infected individuals. However, due to their limited permeability into the brain HIV-1 replication persists in brain reservoirs such as perivascular macrophages and microglia, which cause HIV-1-associated neurocognitive disorders. Therefore, it is highly desirable to find a novel therapy that can cross the blood-brain barrier (BBB) and target HIV-1 pathogenesis in brain reservoirs. A recently developed 2-amino-3-methylpentanoic acid [2-morpholin-4-yl-ethyl]-amide (LM11A-31), which is a p75 neutrotrophin receptor (p75^NTR^) modulator, can cross the BBB. In this study, we examined whether LM11A-31 treatment can suppress HIV-1 replication, oxidative stress, cytotoxicity, and inflammatory response in macrophages. Our results showed that LM11A-31 (100 nM) alone and/or in combination with positive control darunavir (5.5 µM) significantly suppresses viral replication and reduces cytotoxicity. Moreover, the HIV-1 suppression by LM11A-31 was comparable to the HIV-1 suppression by darunavir. Although p75^NTR^ was upregulated in HIV-1-infected macrophages compared to uninfected macrophages, LM11A-31 did not significantly reduce the p75^NTR^ expression in macrophages. Furthermore, our study illustrated that LM11A-31 alone and/or in combination with darunavir significantly suppress pro-inflammatory cytokines including IL-1β, IL-8, IL-18, and TNF-α and chemokines MCP-1 in HIV-induced macrophages. The suppression of these cytokines and chemokines by LM11A-31 was comparable to darunavir. In contrast, LM11A-31 did not significantly alter oxidative stress, expression of antioxidant enzymes, or autophagy marker proteins in U1 macrophages. The results suggest that LM11A-31, which can cross the BBB, has therapeutic potential in suppressing HIV-1 and inflammatory response in brain reservoirs, especially in macrophages.

## Impact statement

The current antiretroviral drugs do not adequately cross the blood-brain-barrier (BBB) and suppress neuroHIV leading to persistent HIV-1 replication in brain reservoirs and subsequently leading to HIV-1-associated neurocognitive disorders (HAND). The efficacy of LM11A-31, a p75^NTR^ modulator, has been extensively reported in several neurological diseases including Alzheimer’s disease (AD), ischemic stroke, and traumatic brain injury. Our study has shown that LM11A-31 can suppress HIV-1 replication in macrophages, and microglia inflammatory functions, which are the major viral reservoirs in the brain. Owing to its ability to cross the BBB, LM11A-31 can potentially be developed as a novel therapy to suppress HIV-1 neuropathogenesis and HAND. Furthermore, since LM11A-31 can potentially treat AD, which is a common comorbidity in HIV-1 aging populations, it can have a dual benefit in HIV-1-AD comorbid conditions.

## Introduction

Human Immunodeficiency Virus- 1 (HIV-1) primarily infects T-cells, peripheral macrophages, and multiple cells in the central nervous system (CNS) including perivascular macrophages, microglia, and astrocytes [1–4]. Combination antiretroviral therapy (ART) has improved the quality of HIV-1-infected individuals by suppressing the virus in the periphery at a controlled level [5]. However, ART drugs poorly cross the blood-brain barrier (BBB) and are unable to suppress the virus in the brain reservoirs, especially perivascular macrophages and microglia [2, 6]. Due to the inability of ART drugs to suppress HIV-1 in the CNS viral reservoirs, HIV-1-associated neurocognitive disorder (HAND) is prevalent among HIV-1-infected individuals [7]. Additionally, many ART drugs have been shown to be neurotoxic [7]. Therefore, it is highly desirable to find safe and effective therapeutic agents, which can effectively cross the BBB and suppress HIV-1 in brain reservoirs.

A recently developed water-soluble compound, 2-amino-3-methylpentanoic acid [2-morpholin-4-yl-ethyl]-amide (LM11A-31), is a small molecule, non-peptide p75^NTR^ ligand, which penetrates the BBB [8]. The compound inhibits the binding of precursor nerve growth factor (proNGF) to the p75 neurotrophin receptor (p75^NTR^). Recent attention has been paid to p75^NTR^ as this receptor is upregulated in several CNS diseases [9]. LM11A-31 is orally bioavailable and no systemic toxicity has been reported [10]. Moreover, LM11A-31 is in phase 2 clinical trials for mild-to-moderate Alzheimer’s disease (AD)^1^ [11]. LM11A-31 activates survival signaling and suppresses degenerative signaling [11]. Recently, the effects of LM11A-31 have been reported in a variety of diseases including but not limited to stroke, AD, Huntington’s disease, and traumatic brain injury [8, 12–14]. Additionally, It has been reported that daily treatment of LM11A-31 for 4 months in HIV-1 gp120 transgenic mice reduces microglial activation and dendritic varicosities, and activates microtubule-associated protein-2 (MAP-2) in the hippocampus [15]. LM11A-31 also decreases cell apoptosis and improves mitochondrial movement in cultured rat neurons following exposure to HIV-1 gp120 or conditioned medium from human monocyte-derived macrophages (MDM), which was treated with gp120 [16]. Treatment of 10 nM LM11A-31 to macrophages and microglia upon exposure to HIV-1 gp120 has been shown to inhibit the release of neurotoxic factors from MDM and microglia. Furthermore, the treatment of 10 nM LM11A-31 reduced or eliminated the neuronal pathology as well as the Feline Immunodeficiency Virus (FIV) effect on astrocytes and microglia [17]. However, FIV replication remained unaffected by LM11A-31. These reports illustrate the promising therapeutic effect of LM11A-31 on HIV-1 pathogenesis. Therefore, we hypothesize that LM11A-31 suppresses HIV-1 pathogenesis including inflammatory functions in HIV-1 infected macrophages.

## Materials and methods

### Cell culture and drug treatment

Human monocyte-derived macrophages (MDM) were prepared as described previously [18]. In brief, peripheral blood mononuclear cells (PBMCs) were obtained from buffy coats (Interstate Blood Bank, Memphis, TN) after density-gradient separation using the established protocol. PBMCs were allowed to adhere to the plastic for 4 h. Then, they were removed and cultured in media supplemented with M-CSF (25 µM) for 7–10 days to promote their differentiation into macrophages. MDM was collected and treated with polybrene for 30 min followed by infection with HIV-1-Ada strain (20 ng/10^6^). The HIV-1 Ada-M monocytetropic virus was obtained from the NIH AIDS Reagent Program (Germantown, MD). Infected cells were seeded in 6-well plates and fresh media was added every third day to maintain cell viability. To determine the p24 antigen levels in the culture supernatant, the collected samples were subjected to p24 ELISA analysis according to the protocol. Once the viral infection is confirmed (7–10 days), HIV-1-infected MDM was treated with either control (DMSO) or LM11A-31 (100 nM). We used DMSO as a control because darunavir (DRV), which was used in subsequent treatments, is dissolved in DMSO.

The HIV-1-uninfected U937 monocytic cell line and HIV-1-infected U1 monocytic cell line were obtained from ATCC (Manassas, VA). The cells were cultured in Roswell Park Memorial Institute (RPMI) 1640 media (Sigma Aldrich, St. Louis, MO), containing 10% FBS (Atlanta biologicals, Atlanta, GA) and 1% L-glutamine (Corning Inc, Tewksbury, MA). To assess the chronic effects of LM11A-31, darunavir (DRV), and combination therapy on HIV-1 replication, we used a previously described method [19]. Briefly, U937 and U1 cells were seeded at 0.4 × 10^6^ cells/mL per well containing 100 nM phorbol-12-myristate-13-acetate (PMA) to differentiate into macrophages and induce HIV-1 replication. At 72h, cells were washed using PBS, and media was substituted with fresh RPMI overnight. The next day, cells were treated once daily using DMSO, LM11A-31 (100 nM), DRV (5.5 μM), and LM11A-31 + DRV (100 nM + 5.5 μM) for 72 h. The supernatant was collected daily to measure the concentration of HIV-1 p24 antigen in U1 cells. Lastly, cells were collected at 24 h following the last dose.

### Measurement of ROS by flow cytometry

The reactive oxygen species (ROS) was measured using 5-(and-6)-chloromethyl-2′,7′-dichlorodihydrofluorescein diacetate, acetyl ester (CM-H2DCFDA), which was obtained from Thermo Fisher Scientific (Waltham, MA). As previously mentioned [20], the level of ROS in DMSO, LM11A-31, DRV, and LM11A-31 + DRV in U1 cells was assessed. In brief, U1 cells were collected and washed using phosphate buffer saline (PBS) after treatment with DMSO or drugs. Then, cells were incubated using the CM-H2DCFDA dye (2–5 µM) for 30 min at 37°C. After incubation, the cells were washed using PBS and the mean fluorescent intensity (MFI) of samples was analyzed using an Agilent Novocyte flow cytometer (Agilent, Santa Clara, CA). The MFI obtained for DMSO-treated control cells served as 100%.

### Western blot analysis

Cells were lysed using RIPA buffer and cellular protein was quantified using Pierce BCA Protein Assay Kit (ThermoFisher Scientific). An equal amount of protein (10 µg) obtained from DMSO- and drug-treated U937 and U1 cells was loaded into polyacrylamide gel and electrophoresed, followed by transfer to a PVDF membrane. Then, the membrane was blocked using Li-Cor blocking buffer (LI-COR Biosciences, Lincoln, NE) for 1h. Then, membranes were incubated overnight at 4°C with primary antibodies, including p75^NTR^ (1:500, catalog no. sc-271708, Santa Cruz Biotechnology), SOD1 (1:500, catalog no. sc-101523, Santa Cruz Biotechnology), Catalase (1:500, catalog no. sc-365738, Santa Cruz Biotechnology), Beclin-1 (1:500, catalog no. sc-11427, Santa Cruz Biotechnology), p-Akt (1:1,000, catalog no. S473, Cell Signaling). and β-Actin (1:1,000, catalog no. 3700, Cell Signaling). The next day, membranes were washed three times for 5 min each in PBS containing 0.2% Tween 20 and then incubated with either goat anti-mouse or goat anti-rabbit secondary antibody (1:10,000 dilution, LI-COR Biosciences) for 1 h at room temperature in the dark. Membranes were washed again and scanned using the Li-Cor Scanner (LI-COR Biosciences) for 1h following the secondary antibody. Densitometry analyses of the proteins were conducted using LI-COR Image Studio Software (v.5.2, Nebraska, United States).

### Multiplex ELISA

As previously reported [21], the level of cytokines, both pro-inflammatory and anti-inflammatory, and chemokines from cells supernatants were evaluated using customized human 9-Plex ProcartaPlex™ multiplex immunoassay (ThermoFisher Scientific). In brief, U937 and U1 cells were treated with DMSO, LM11A-31, DRV, and LM11A-31 + DRV for 24 and 48 h. Then, supernatants from U937 and U1 cells were collected at 24 and 48 h and transferred to an ELISA plate. Following incubation of samples and standards using a magnetic 96-well ELISA plate for 1 h at room temperature, wells were washed, and then detection antibody, streptavidin-PE, and reading buffer were added. According to the manual, the levels of cytokines/chemokines quantification were measured. The concentration of cytokines and chemokines was reported as pg/mL.

### HIV-1 type 1 p24 ELISA

In this study, we used HIV-1 p24 ELISA to measure HIV-1 replication. The majority of HIV-1 p24 assays available in the market utilize a conventional ELISA to capture and identify the p24 antigen. These techniques can detect HIV-1 p24 antigen in the range of 5–25 pg/mL without the need for signal amplification [22, 23]. The HIV-1 Type 1 p24 Antigen ELISA kit was purchased from ZeptoMetrix Corporation (Buffalo, NY). The level of HIV-1 p24 antigen in supernatant collected from U1 macrophages (24, 48, and 72 h post-PMA treatment) and HIV-1-infected MDM (24 and 48 h) was assessed using the enzyme-linked immunoassay (HIV-1 p24 ELISA). Viral antigen in the media is captured into the immobilized monoclonal antibody for p24 coated on microwells in the p24 assay. According to the manufacturer’s protocol, the captured viral antigen was sequentially exposed to a biotin-labeled human antibody to HIV-1, streptavidin conjugated to horseradish peroxidase, and tetramethylbenzidine substrate. The optical density of each well was analyzed using a microplate reader at 450 nm and compared against the standard curve to find the concentration of HIV-1 p24 antigen (pg/mL) in the samples. The amount of HIV-1 p24 from DMSO-treated U1 cells was treated as 100%.

### LDH activity

To assess the cytotoxic effect of DMSO, LM11A-31, DRV, and combination therapy in U937 and U1 cells, the CyQUANT™ LDH Cytotoxicity Assay Kit (Catalog no. C20300, ThermoFisher Scientific) was used. In brief, U937 and U1 cells were treated using DMSO or drugs for 24, 48, and 72 h. Then, supernatants were collected daily and transferred to a 96-well plate. The reaction mixture was added to the cell culture supernatant for 30 min at room temperature. LDH activity was analyzed using a microplate reader at 680 and 490 nm. Background absorbance of 680 nm was subtracted from 490 nm absorbance to find the LDH activity.

### Statistical analysis

The results were expressed as mean ± SEM. Differences among experimental groups were assessed by student’s t-test or one-way ANOVA followed by Tukey’s *post hoc* test. The level of significance was set at *p < 0.05, **p < 0.01, ***p < 0.001, ****p < 0.0001.

## Results

### The effect of LM11A-31 on cytotoxicity, antioxidant enzymes, and autophagy marker in HIV-1-uninfected U937 macrophages

**FIGURE 1:**
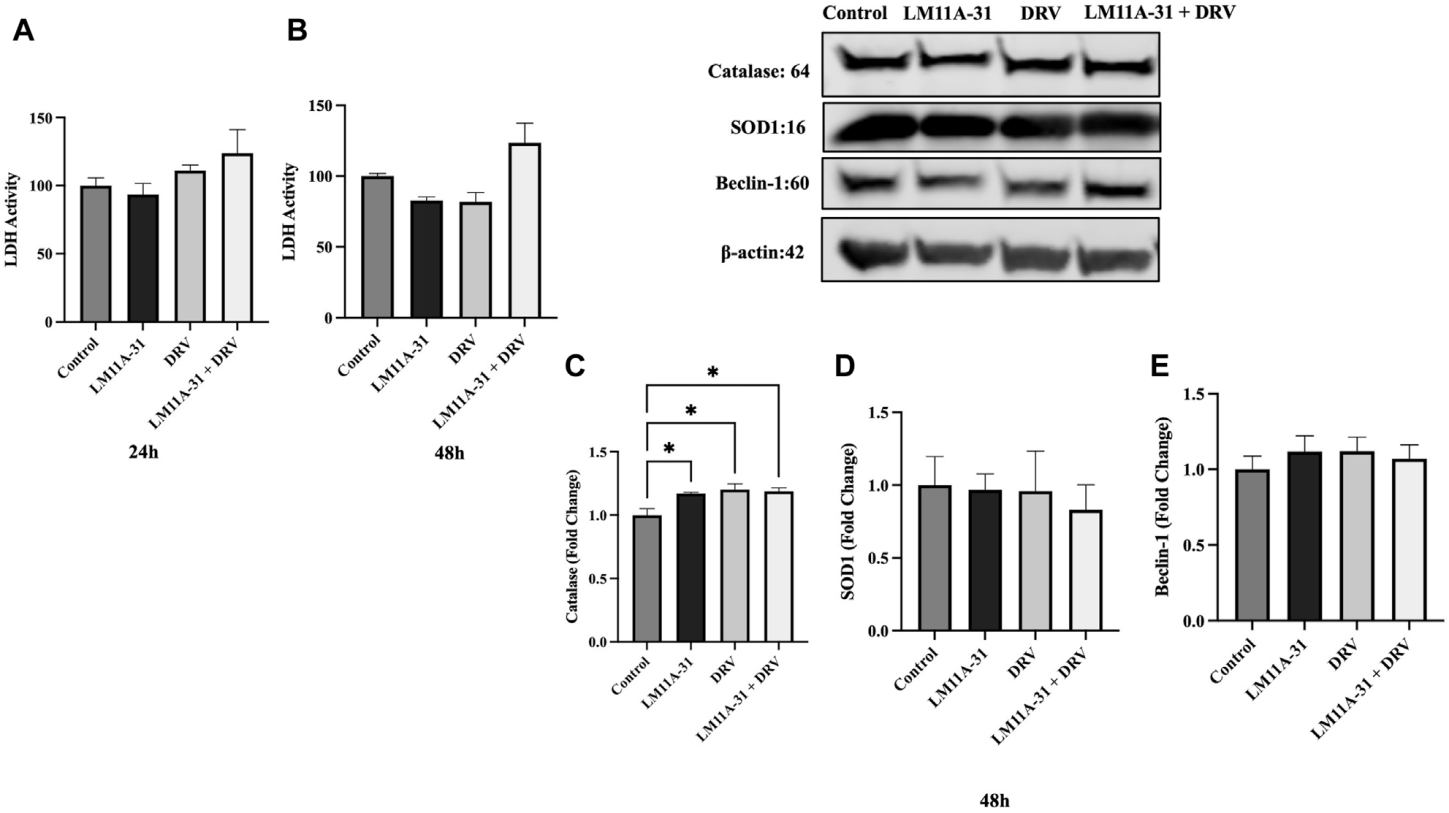
The effect of LM11A-31 on cytotoxicity, antioxidant enzymes, and autophagy marker in U937 macrophages. U937 macrophages were differentiated and then treated with DMSO, LM11A-31 (100 nM), DRV (5.5 μM), and LM11A-31 + DRV for 48 h. The cytotoxicity was measured using CyQUANT™ LDH Cytotoxicity Assay Kit. **(A)** LDH measurement at 24 h. **(B)** LDH measurement at 48 h. **(C)** Quantification of protein level of catalase at 48 h. **(D)** Quantification of protein level of SOD1 at 48 h. **(E)** Quantification of protein level of Beclin-1 at 48 h. Values are expressed as mean ± SEM (n = 3), *p < 0.05.

Before assessing the effect of LM11A-31 in MDM and HIV-1-infected U1 macrophages, we determined whether LM11A-31 is cytotoxic to HIV-1-uninfected U937 macrophages (control cells). U937 macrophages were treated with LM11A-31 (100 nM), DRV, and LM11A-31 + DRV at 24 and 48 h. The results showed that LM11A-31 and DRV are not cytotoxic at 24 and 48 h in U937 macrophages (Figures 1A, B). Furthermore, we determined whether LM11A-31 alters antioxidant enzymes, SOD1 and catalase (markers of oxidative stress), and Beclin-1, a marker of autophagy. Our results demonstrated that LM11A-31, DRV, and combination therapy do not alter the protein levels of catalase (Figure 1C), SOD1 (Figure 1D), and Beclin-1 (Figure 1E) at 24 and 48 h in U937 macrophages.

### The effect of LM11A-31 on inflammatory response in U937 macrophages

**FIGURE 2:**
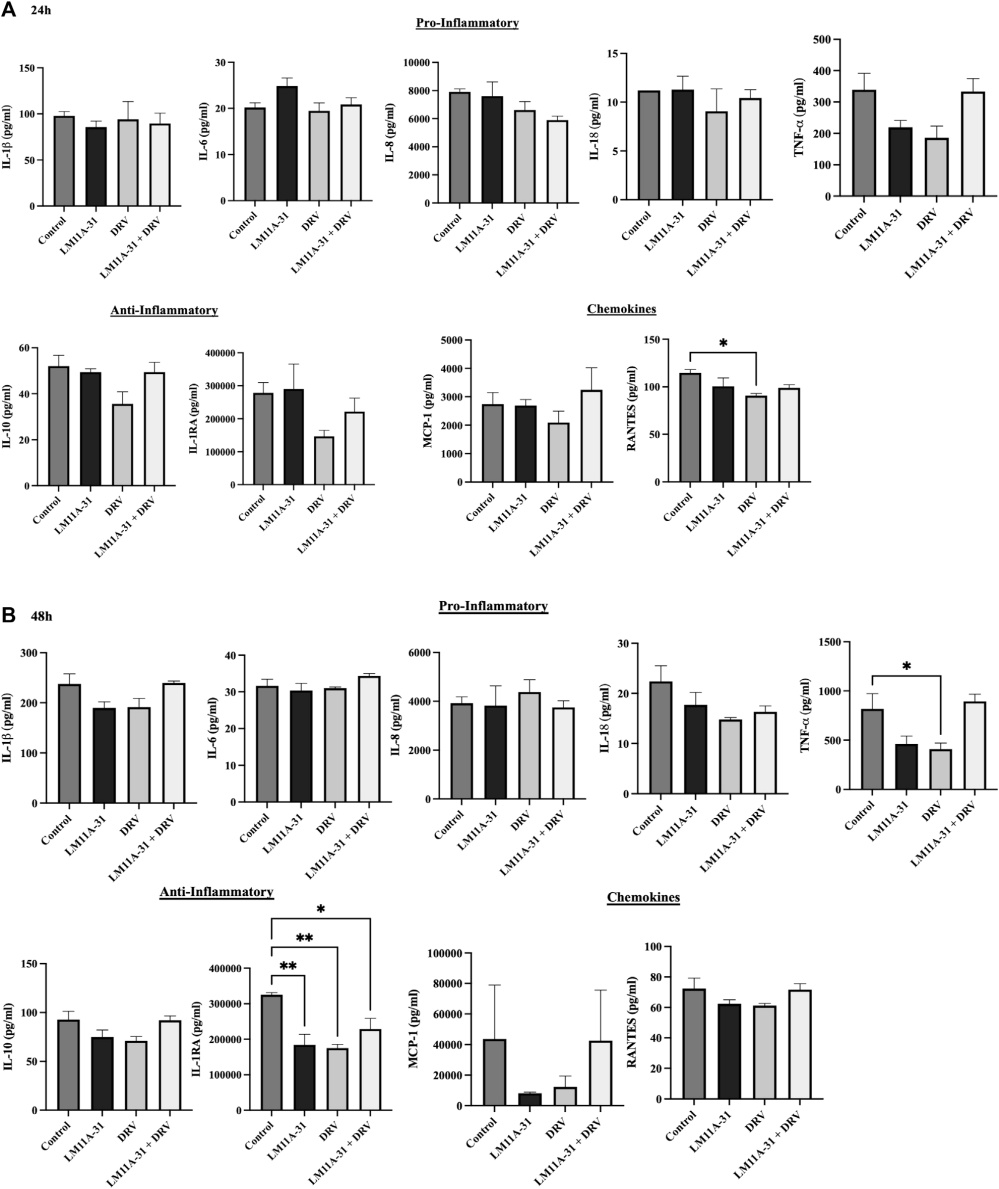
The effect of LM11A-31 on inflammatory response in U937 macrophages. U937 macrophages were differentiated and then treated with DMSO, LM11A-31 (100 nM), DRV (5.5 μM), and LM11A-31 + DRV for 48 h. The cytokines and chemokines profile were measured using a customized human 9-Plex ProcartaPlex™ multiplex immunoassay. **(A)** Proinflammatory, chemokines, and anti-inflammatory measurement at 24 h. **(B)** Proinflammatory, chemokines, and anti-inflammatory measurement at 48 h. Values are expressed as mean ± SEM (n = 3). *p < 0.05, **p < 0.01.

To evaluate whether LM11A-31 affects inflammatory response in the control U937 macrophages, the cells were treated with LM11A-31, DRV, and LM11A-31 + DRV at 24 and 48 h. We then conducted cytokine assays to measure the levels of key pro-inflammatory, and anti-inflammatory cytokines, and chemokines. The data demonstrated that LM11A-31 and DRV do not alter the levels of pro-inflammatory and anti-inflammatory cytokines at 24 h (Figure 2A). Moreover, no changes were observed in the levels of pro-inflammatory cytokines and chemokines at 48 h by LM11A-31 (Figure 2B). However, DRV significantly reduced the levels of RANTES at 24 h (Figure 2A) and TNF-α at 48 h (Figure 2B). Furthermore, LM11A-31, DRV, and combination therapy significantly decreased the level of IL-1RA at 48 h (Figure 2B). Overall, the results suggest that LM11A-31 does not show a significant inflammatory response in U937 macrophages.

### The effect of LM11A-31 on viral replication in MDM

**FIGURE 3:**
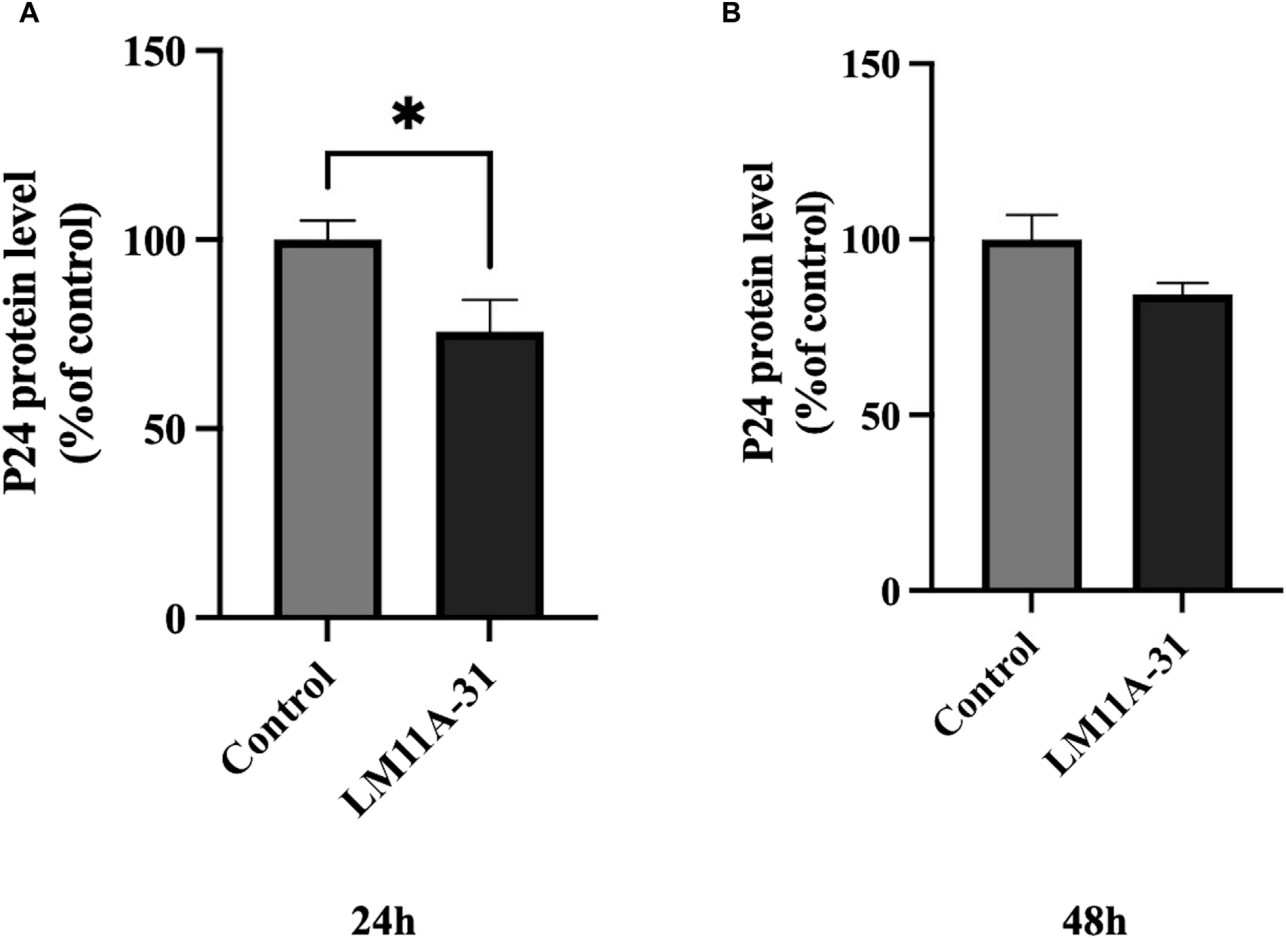
The effect of LM11A-31 on HIV-1 replication in MDM. MDM was prepared and infected with HIV-1 as described in Materials and Methods. MDM was treated with DMSO and LM11A-31 (100 nM) for 48 h. The HIV-1 replication was measured using p24 ELISA kit. **(A)** P24 measurement at 24 h. **(B)** P24 measurement at 48 h. Values are expressed as mean ± SEM (n = 3), *p < 0.05.

We examined whether LM11A-31 can suppress the viral replication in MDM following 24 and 48 h of acute treatment by measuring the protein level of p24. We found that LM11A-31 significantly decreased (*p* < 0.05) viral replication compared to the control at 24 h treatment (Figure 3A). In contrast, LM11A-31 did not result in significant change at 48 h treatment (Figure 3B). Following confirmation that LM11A-31 is potentially efficacious in suppressing HIV-1 in primary macrophages, we used HIV-1-infected U1 macrophages for the subsequent studies because the U1 monocytic or HIV-1-infected U937 cell line is the standard model to study HIV-1 pathogenesis [24].

### The effect of LM11A-31 on viral replication in U1 macrophages

**FIGURE 4:**
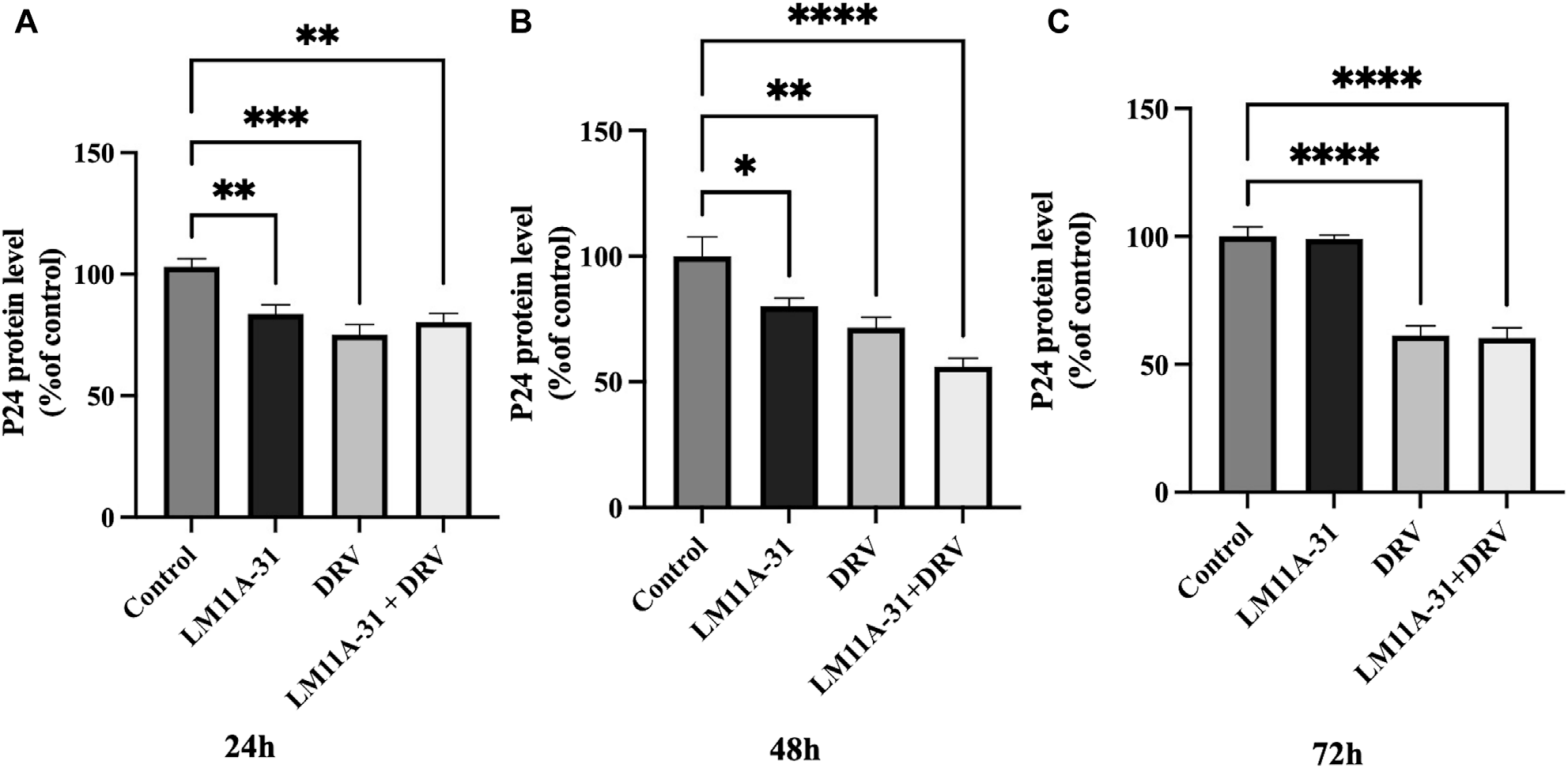
The effect of LM11A-31 on HIV-1 replication in U1 macrophages. U1 macrophages were differentiated and then treated with DMSO, LM11A-31 (100 nM), DRV (5.5 μM), and LM11A-31 + DRV for 72 h. The HIV-1 replication was measured using p24 ELISA kit. **(A)** P24 measurement at 24 h. **(B)** P24 measurement at 48 h. **(C)** P24 measurement at 72 h. Values are expressed as mean ± SEM (n = 3–6), *p < 0.05, **p < 0.01, ***p < 0.001, ****p < 0.0001.

To examine the effect of LM11A-31, a positive control DRV, and combination on HIV-1 replication in U1 macrophages, we treated the U1 cells at 24, 48, and 72 h. The results showed that LM11A-31 (100 nM) and positive control DRV (5.5 μM) significantly (*p* < 0.05) suppressed the viral replication following 24 and 48 h treatments in U1 macrophages (Figures 4A, B). Interestingly, the HIV-1 suppression by both LM11A-31 and positive control DRV was comparable at both 24 and 48 h treatments. Although both DRV and the combination showed a significant reduction, LM11A-31 did not show a significant change in the viral replication following 72 h treatment (Figure 4C). LM11A-31 may be less stable, or partially metabolized or effluxed out in U1 macrophages at 72 h.

### The effect of LM11A-31 on cytotoxicity in U1 macrophages

**FIGURE 5:**
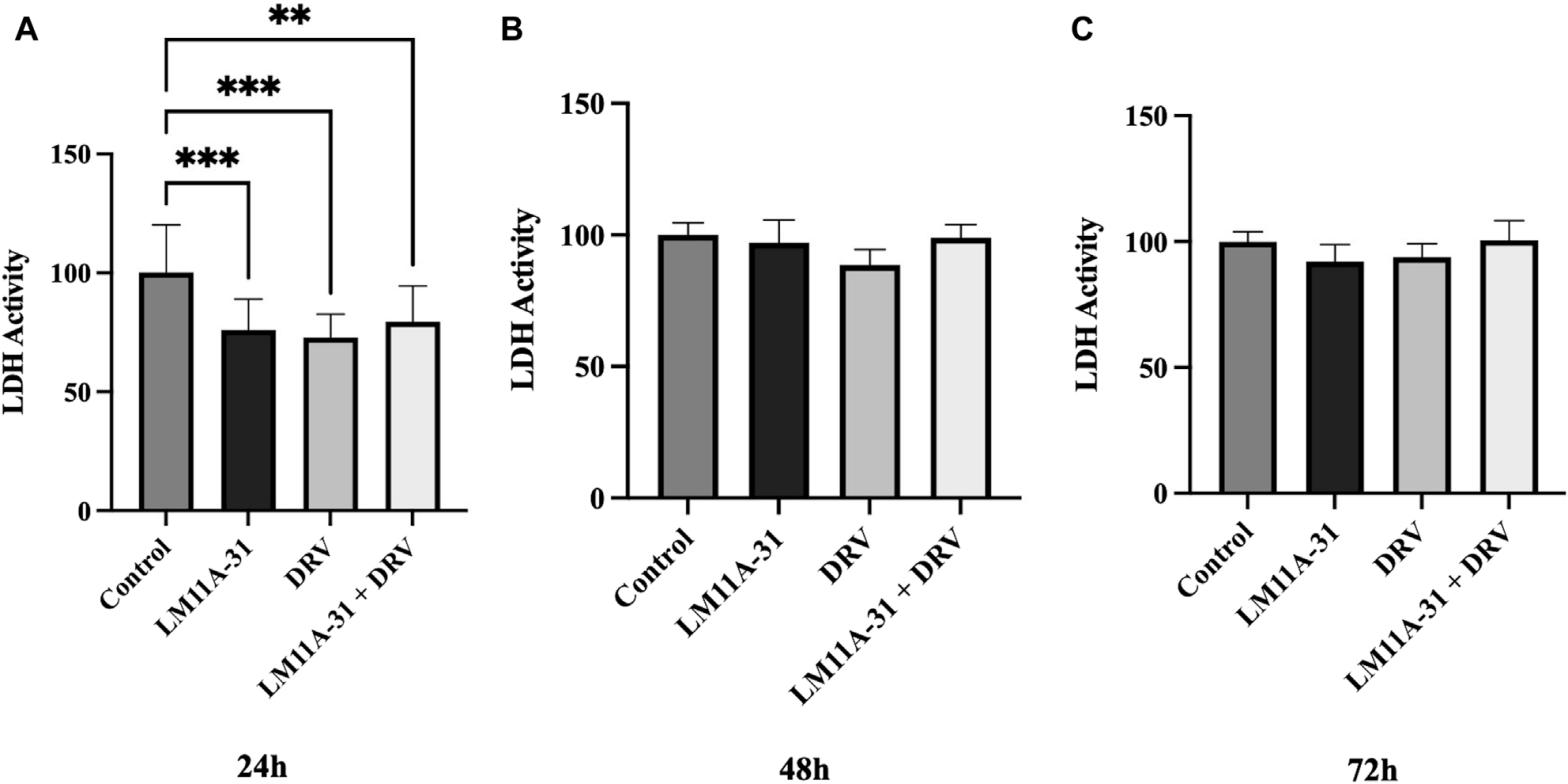
The effect of LM11A-31 on cytotoxicity in U1 macrophages. U1 macrophages were differentiated and then treated with DMSO, LM11A-31 (100 nM), DRV (5.5 μM), and LM11A-31 + DRV for 72 h. The cytotoxicity was measured using CyQUANT™ LDH Cytotoxicity Assay Kit. **(A)** LDH measurement at 24 h. **(B)** LDH measurement at 48 h. **(C)** LDH measurement at 72 h. Values are expressed as mean ± SEM (n = 3–6), *p < 0.05, **p < 0.01, ***p < 0.001.

To examine the effect of LM11A-31, DRV, and combination therapy on cytotoxicity in U1 macrophages, we treated U1 cells at 24 h, 48 h, and 72 h and then assessed cytotoxicity using the LDH assay. Our results showed that LM11A-31, DRV, and combination therapy significantly (*p* < 0.05) reduced cytotoxicity compared to the control group following 24 h treatments (Figure 5A). However, no effect on cytotoxicity was observed after 48 and 72 h treatments in U1 macrophages (Figures 5B, C).

### The effect of HIV-1 infection in U1 macrophages on p75^NTR^ regulation

**FIGURE 6:**
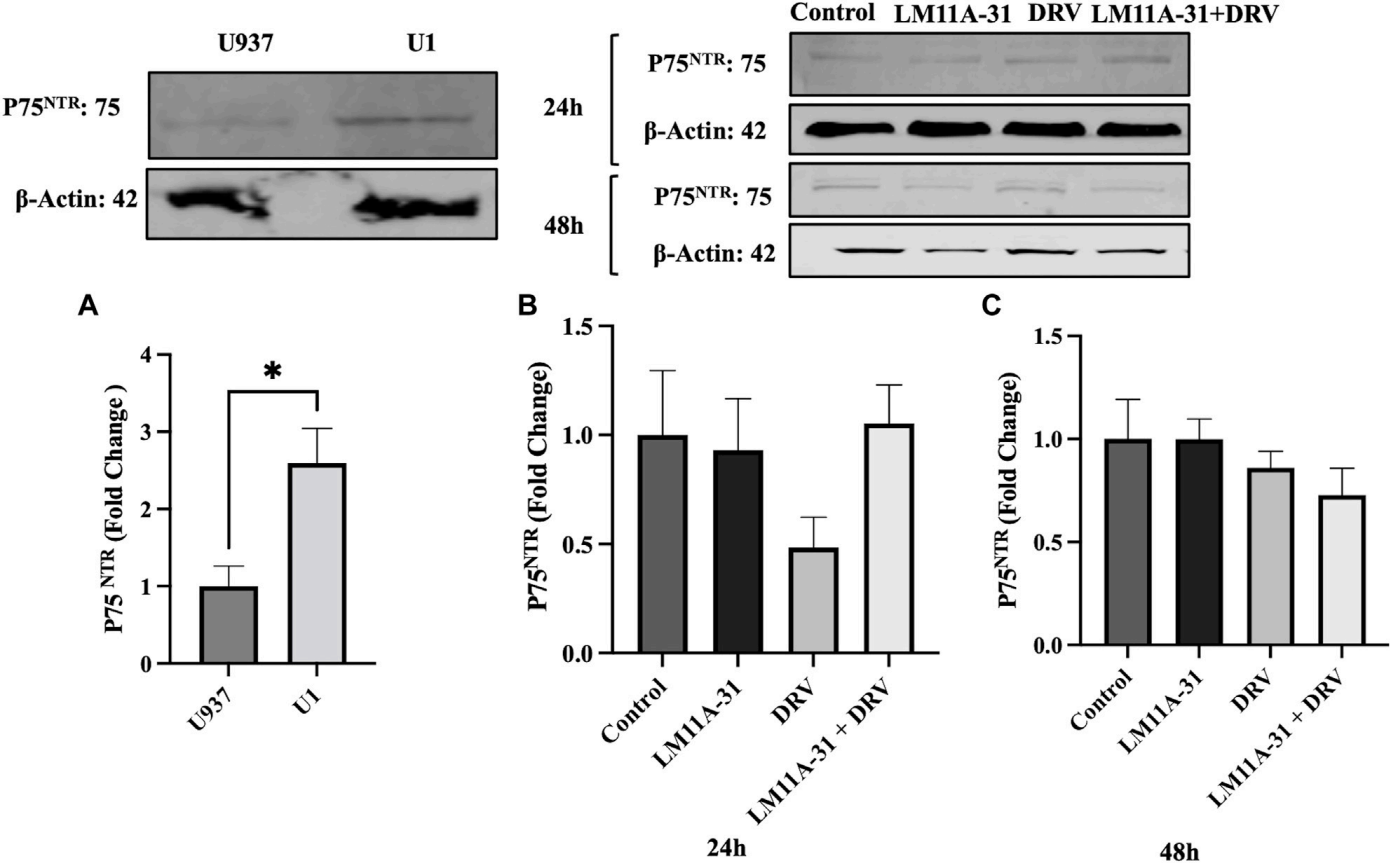
The effect of p75^NTR^ in LM11A-31-mediated viral replication in U1 macrophages. U1 macrophages were differentiated and then treated with DMSO, LM11A-31 (100 nM), DRV (5.5 μM), and LM11A-31 + DRV for 48 h. The protein expression was measured using a western blot. **(A)** Quantification of protein level of p75^NTR^ in U1 and U937. **(B, C)** Quantification of protein level of p75^NTR^ at 24 and 48 h. Values are expressed as mean ± SEM (n = 3–4), *p < 0.05.

To explore whether p75^NTR^ is altered in HIV-1-infected cells, we seeded U1 and U937 (uninfected) macrophages and then measured the protein level of p75^NTR^ using western blotting. We found that p75^NTR^ expression was significantly upregulated (*p* < 0.05) in U1 compared to U937 macrophages (Figure 6A). This result indicates that HIV-1 infection stimulates protein expression of p75^NTR^. We then determined whether LM11A-31 and/or DRV suppress p75^NTR^. Our western blot, data showed that treatment with LM11A-31 and/or DRV does not significantly downregulate p75^NTR^ expression in U1 macrophages at 24 h (Figure 6B). However, there was a trend in the reduction in the level of p75^NTR^ after treatment at 48 h (Figure 6C).

### The effect of LM11A-31 on autophagy and cell survival pathways in U1 macrophages

**FIGURE 7:**
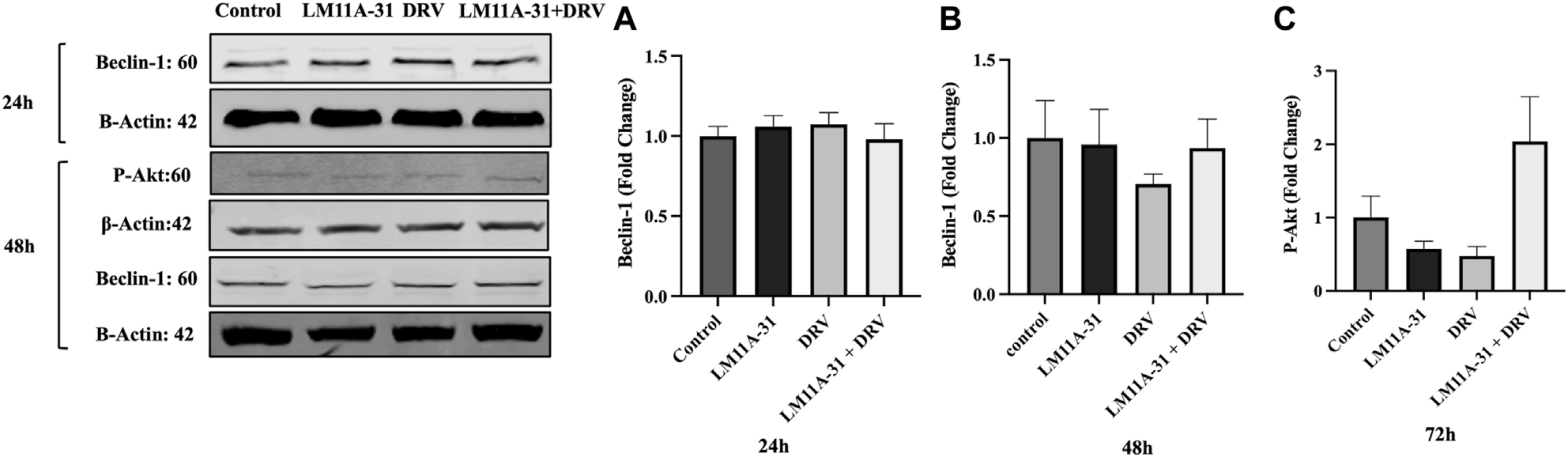
The effect of LM11A-31 on autophagy and cell survival pathways in U1 macrophages. U1 macrophages were differentiated and then treated with DMSO, LM11A-31 (100 nM), DRV (5.5 μM), and LM11A-31 + DRV for 48 h. The protein expression was measured using a western blot. **(A, B)** Quantification of protein level of Beclin-1 at 24 and 48 h. **(C)** Quantification of protein level of p-Akt at 48 h.Values are expressed as mean ± SEM (n = 4).

ER stress followed by autophagic dysregulation plays a significant role in the pathogenesis of HIV-1 infection [25]. To determine the role of LM11A-31 on autophagic dysregulation, we performed a western blot for the autophagy marker protein, Beclin-1 in U1 macrophages following 24 and 48 h treatments. Our results showed that LM11A-31 does not change the protein level of Beclin-1 compared to the control at both time points (Figures 7A, B). Further, treatment using DRV alone and in combination with LM11A-31 did not affect the expression of Beclin-1. Since p75^NTR^ has been shown to be involved in cell survival via the p-Akt pathway, we decided to examine whether LM11A-31 alters the protein level of p-Akt in U1 macrophages. However, no change was observed in the protein level of p-Akt following LM11A-31 alone and/or in combination with DRV at 48 h treatment (Figure 7C).

### The effect of LM11A-31 on oxidative stress in U1 macrophages

**FIGURE 8:**
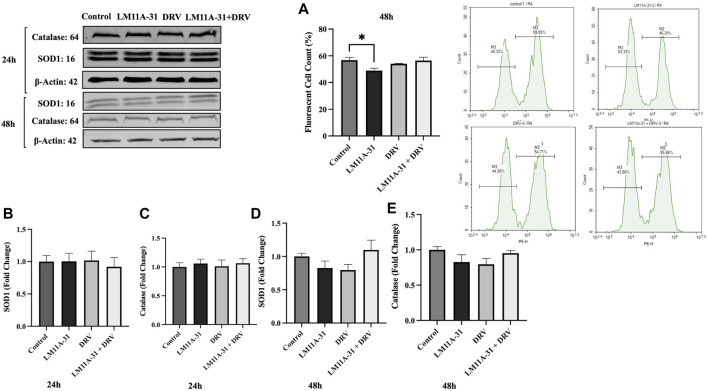
The effect of LM11A-31 on oxidative stress in U1 macrophages. U1 macrophages were differentiated and then treated with DMSO, LM11A-31 (100 nM), DRV (5.5 μM), and LM11A-31 + DRV for 48 h. The ROS was measured using flow cytometry. The protein expression was measured using a western blot. **(A)** Measurement of ROS at 48 h, *p < 0.05. **(B)** Quantification of protein level of SOD1 at 24 h. **(C)** Quantification of protein level of catalase at 24 h. **(D)** Quantification of protein level of SOD1 at 48 h. **(E)** Quantification of protein level of catalase at 48 h. Values are expressed as mean ± SEM (n = 3–4).

Oxidative stress is one of the major factors in HIV-1 pathogenesis. Therefore, we determined whether LM11A-31 alters ROS and antioxidant enzymes protein level, SOD1, and catalase. For this, U1 macrophages were treated using DMSO, LM11A-31, DRV, and combination therapy for 48 h, and ROS and antioxidant enzymes were assessed. Our results showed that LM11A-31 significantly reduced ROS in U1 macrophages following 48 h treatment Additionally, DRV and combination therapy did not alter the ROS level compared to the control group (Figure 8A). Further, the levels of SOD1 and catalase were also unaltered following treatment of LM11A-31, DRV, and combination at 24 h (Figures 8B, C) and 48 h treatments (Figures 8D, E). Overall, the results showed that LM11A-31 either alone or in combination with DRV did not enhance oxidative stress. This is important because many ART drugs and/or drug combinations are known to increase oxidative stress [26, 27].

### The effect of LM11A-31 on inflammatory response in U1 macrophages

**FIGURE 9:**
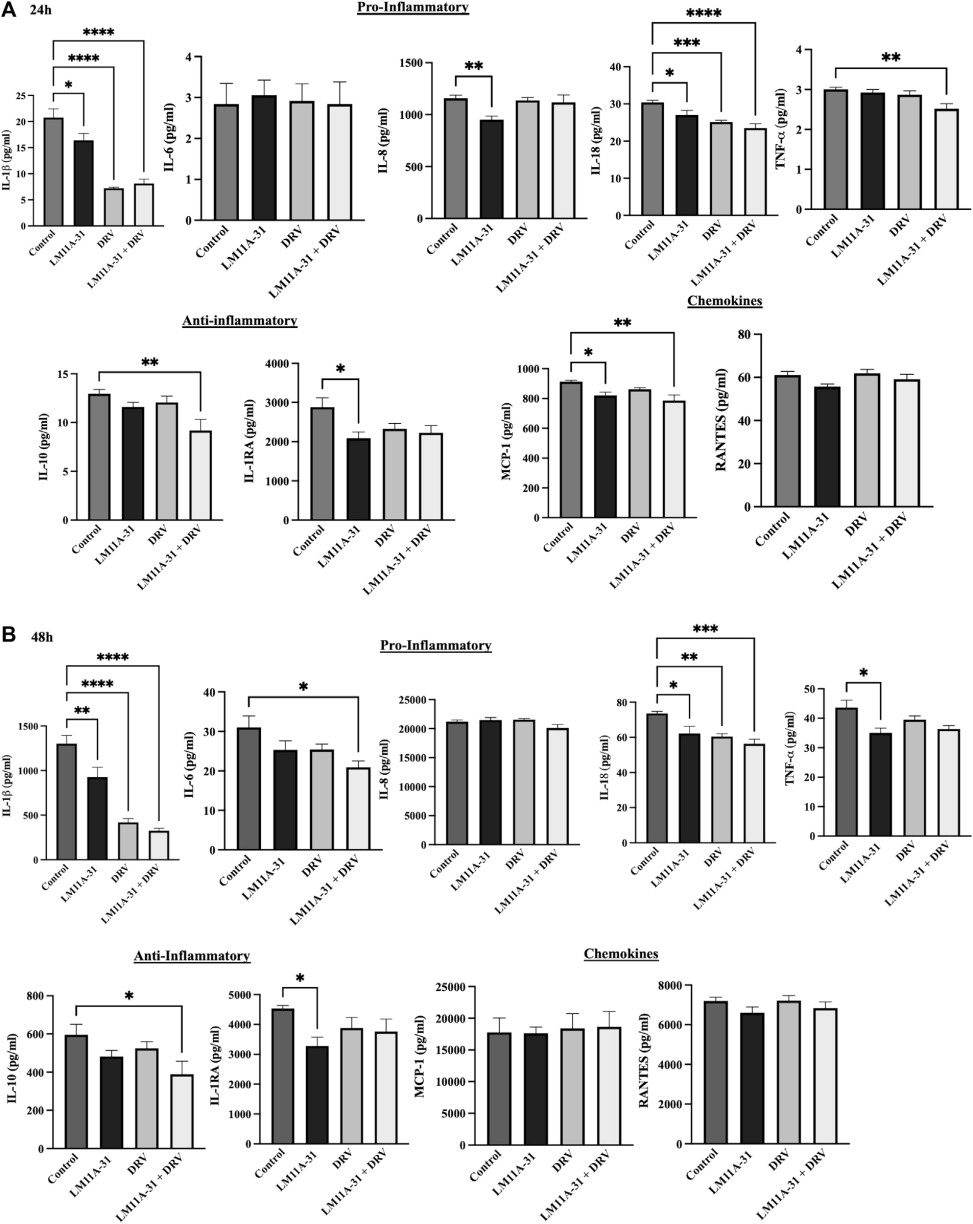
The effect of LM11A-31 on inflammatory response in U1 macrophages. U1 macrophages were differentiated and then treated with DMSO, LM11A-31 (100 nM), DRV (5.5 μM), and LM11A-31 + DRV for 48 h. The cytokines and chemokines profile were measured using a customized human 9-Plex ProcartaPlex™ multiplex immunoassay. **(A)** Proinflammatory, chemokines, and anti-inflammatory measurement at 24 h. **(B)** Proinflammatory, chemokines, and anti-inflammatory measurement at 48 h. Values are expressed as mean ± SEM (n = 4–6). *p < 0.05, **p < 0.01, ***p < 0.001, ****p < 0.0001.(b)

Inflammation plays a significant role in the pathogenesis of HIV-1. To determine the impact of LM11A-31 on inflammation, we performed a cytokine assay for the major HIV-1-associated pro-inflammatory and anti-inflammatory cytokines and chemokines on U1 macrophages following 24 and 48 h treatments. (Figures 9A, B). For this, we ran a series of pro-inflammatory (IL-1β, IL-6, IL-8, IL-18, and TNF-α), anti-inflammatory cytokines (IL-1RA and I-10), and chemokines (MCP-1 and RANTES) upon treatments with LM11A-31, DRV, and combination at 24 and 48 h treatments. The results showed that treatment of U1 macrophages using LM11A-31 significantly reduced the levels of IL-1β, IL-8, and IL-18 compared to the control at 24 and 48 h (Figures 9A, B; *p* < 0.05). Additionally, DRV significantly reduced the levels of IL-1β and IL-18 at both time points (Figures 9A, B). Combination therapy using LM11A-31 and DRV also significantly downregulated IL-1β, IL-18, and TNF-α at 24 and 48 h (Figures 9A, B; *p* < 0.05). However, the level of anti-inflammatory cytokines IL-1RA, and IL-10, were also significantly reduced in LM11A-31 + DRV and LM11A-31 groups compared to the control at 24 and 48 h, respectively (Figures 9A, B; *p* < 0.05). With regards to proinflammatory chemokine, the level of MCP-1 was significantly reduced in LM11A-31 and LM11A-31 + DRV groups compared to the control at 24 h (Figure 9A; *p* < 0.05). No changes were observed in the level of RANTES for both time points (Figures 9A, B). Overall, the results with LM11A-31 on reducing inflammatory cytokines were comparable to the results obtained by the known ART drug, DRV.

## Discussion

Despite freely and widely available ART drugs for HIV-1 treatment, they are unable to suppress the HIV-1 replication in the brain reservoirs. Macrophages express the entry receptor CD4 and co-receptors CCR5 and CXCR4 which interact with the envelope protein of HIV-1 to facilitate entry and infection. They are viral reservoirs and represent a source of infection [28]. In contrast to T cells, productive HIV-1 infection happens in macrophages without relying on the synthesis of cellular DNA, and ART drugs exhibit distinct mechanisms in macrophages and lymphocytes [29]. Pro-inflammatory cytokines have a significant impact on HIV-1 pathogenesis in macrophages [30].

This is the first report that showed that the modulator of p75^NTR^, LM11A-31, suppresses viral replication and reduces cytotoxicity and inflammatory response in macrophages. LM11A-31 can cross the BBB and suppress the virus in CNS reservoirs. Previous reports have shown the potential role of LM11A-31 in multiple neuronal diseases by modulating p75^NTR^ [15–17]. In this study, we observed that HIV-1-infected U1 macrophages upregulate the expression of p75^NTR^ compared to the uninfected U937 macrophages. Our results are consistent with previous reports, which showed that HIV-1 protein gp120 activates p75^NTR^ expression [15, 31]. Although there is a trend, a lack of significant reduction in the level of p75^NTR^ by LM1A-31 in U1 macrophages suggests that LM11A-31 likely suppresses HIV-1 via multiple pathways.

The current study showed that the effect of LM11A-31 is comparable to the effect caused by positive control DRV. We have previously shown that DRV significantly reduces p24 viral replication in HIV-1-infected monocytic cells including U1 macrophages [32, 33]. HIV-1 RNA, anti-HIV antibodies, and HIV-1 capsid protein (p24 antigen) serve as a primary viral indicator for the identification of HIV-1 infection and for monitoring the advancement of the disease [22]. The p24 antigen becomes detectable approximately 2 weeks following HIV-1 infection due to the initial rapid viral replication phase, which is correlated with viremia elevation.

Our results clearly showed that LM11A-31 suppresses viral replication in both MDM and U1 macrophages, especially at early time points, in acute treatment. It can also be noted that while in MDM its effect is diminished after 24 h, in U1 macrophages the effect continues until 48 h. Although results obtained from U1 macrophages have been replicated in MDM [18, 34], it is possible that MDM is more sensitive to LM11A-31 exposures than U1 macrophages. Primary macrophages and U1 macrophages might have differences in cellular mechanisms and metabolic activities, which can lead to the different effects of LM11A-31 on viral replication [35, 36]. Furthermore, macrophages have different activation states including, M1 or M2 polarization, which may be differentially affected by various drug exposures including LM11A-31 in MDM and U1 macrophages [37, 38].

Further, our results clearly show that the LM11A-31 effect continues for a relatively short period compared to DRV, which sustains the effects until 72 h in U1 macrophages. The findings can be explained by the fact that LM11A-31 is hydrophilic, while DRV is a lipophilic drug [8, 39]. Being hydrophilic, it may be metabolized or destabilized more rapidly than DRV. The findings are also consistent with the literature that LM11A-31 has a relatively lower half-life than DRV (3–4 h vs. 7 h) [10]. LM11A-31 also showed additive effects with DRV at 48 h treatment in U1 macrophages. These findings have significant clinical relevance because LM11A-31 can cross the BBB. Moreover, LM11A-31 has shown the potential to treat several neurological diseases, especially AD, which is a common comorbidity in HIV-1 aging populations [8, 11, 14].

Another interesting observation was that LM11A-31 showed relatively lower cytotoxicity compared to the control and compared to DRV, at 24h in U1 macrophages. Literature has shown that several ART drugs are cytotoxic in brain cells including HIV-1 reservoirs macrophages, microglia, and astrocytes [40]. As expected, LM11A-31 and DRV did not trigger cytotoxicity in control U937 macrophages at 24 and 48 h.

Our study has shown that DRV in combination with ritonavir reduces cell viability [41]. Thus, LM11A-31, being less toxic, could be potentially added with ART treatment to suppress HIV-1 in brain reservoirs. This would also reduce the ART drug load and ART-induced neurotoxicity.

Oxidative stress is one of the hallmarks of HIV-1 pathogenesis, especially in CNS [42]. In our previous report, we have shown that several factors including cigarette smoking trigger oxidative stress and contribute to HIV-1 replication [18, 43]. The present study exhibits that LM11A-31 suppresses ROS in U1 macrophages. While the protein expression of two important antioxidant stress enzymes (SOD1 and catalase) was not altered by LM11A-31 treatment after 24 and 48 h. It is possible that these enzymes in LM11A-31-treated U1 macrophages are at optimum levels to reduce the ROS levels. Additionally, LM11A-31 and DRV did not change the protein expression of these enzymes in U937 macrophages at 48 h, suggesting that LM11A-31 does not cause oxidative stress. Importantly, unlike many drugs including ART drugs [26, 27], LM11A-31 did not increase oxidative stress. Our previous study has shown that DRV in combination with ritonavir enhances oxidative stress by enhancing the levels of ROS and reducing SOD1 [41].

Inflammation is another hallmark of HIV-1 pathogenesis, especially in brain reservoirs [44]. Moreover, recently we have shown that benzo(a)pyrene, which is a major component of cigarettes, induces IL-1β and other cytokines and chemokines in U1 macrophages [45]. Furthermore, we have shown that IL-1β released from HIV-infected U1 macrophages are taken up by neuronal cells leading to neuroinflammation [46]. This phenomenon occurs via extracellular vesicles (EVs) mediated intercellular interactions. Like viral proteins, the inflammatory agents can also be taken up by neurons directly or by packaging in EVs and modulate neuronal inflammation [47]. In this context, cytokines, and chemokines, released from macrophages, can be packaged in EVs, and delivered to neurons reducing neuroinflammation. Therefore, drugs that reduce inflammation in macrophages or neuronal cells are important in reducing HIV-1-induced neuroinflammation. Our results exhibited that LM11A-31 alone and in combination with DRV downregulates many pro-inflammatory cytokines IL-1β, IL-8, IL-18, and TNF-α and chemokine MCP-1. Our results are consistent with the previous report that HIV-1-infected monocytes increase the production of pro-inflammatory cytokines [48]. Importantly, LM11A-31 or DRV did not alter the levels of most pro-inflammatory cytokines and chemokines in U937 macrophages, except for an anti-inflammatory cytokine IL-1RA at 48 h. Taken together, our findings indicate that LM11A-31 reduces HIV-1-induced inflammatory response.

It has been reported that HIV-1 infection increases the level of IL-10 and suppression of IL-10 improves the function of T cells in HIV-1 patients [49]. IL-1RA suppresses IL-1-induced viral replication in U1 macrophages [50]. IL-1 promotes viral replication through IL-1RA [50]. These data demonstrate that LM11A-31 suppresses inflammatory cytokines alone or along with DRV in U1 macrophages. ART drugs, especially protease inhibitors, show increased levels of proinflammatory cytokines in the serum of HIV-1-infected individuals who were treated with different ART drugs [51]. Thus, the addition of LM11A-31 could work effectively as an additive agent with ART drugs without causing drug-induced inflammation.

ER, stress and subsequent autophagic dysregulation by ART drugs are other characteristics of HIV-1 pathogenesis, especially in macrophages [7, 52]. In a study, it has been shown that the ART drug efavirenz dysregulates autophagy by blocking the activity of the Beclin-1/Atg14/PI3KIII complex [53]. Therefore, we determined whether LM11A-31 alone or in combination with DRV also dysregulates autophagy by measuring the levels of Beclin-1. The inability of LM11A-31 to alter the levels of belclin-1 suggests that unlike efavirenz, LM11A-31 is safe with regards to autophagic activity and subsequent autophagic cell death.

A study from Meeker’s group in FIV, which is like HIV-1 in producing systemic and CNS disease, has demonstrated the role of LM11A-31 in reducing FIV-induced microglial, astrocytic, and neuronal pathogenesis [17]. LM11A-31 prevented the development of foci of calcium accumulation and beading in the dendrites in feline neurons upon exposure to a conditioned medium from FIV-treated macrophages. The findings from Meeker’s study suggest that LM11A-31 may have excellent potential for the treatment of HIV-1-associated neurodegeneration [17]. Our findings from LM11A-31 in U1 macrophages are somewhat consistent with Meeker’s FIV study on neuronal pathogenesis. For example, our study suggests that LM11A-31 has the potential to reduce not only HIV-1 replication in myeloid-derived cells (e.g., U1 macrophages) but also oxidative stress and inflammation, which are known to cause HIV-associated neuropathogenesis, including HAND.

Previous reports have shown that HIV-1-infected individuals are susceptible to developing many other neuronal diseases/conditions including cognitive disorders, stroke, AD, and rapid aging in their life [54]. A phase 2a safety and exploratory endpoint trial in AD subjects testing LM11A-31 has recently been completed (EU Clinical Trials Registration: 2015-005263-16, ClinicalTrials.gov registration: NCT03069014). Furthermore, our group found the efficacy of LM11A-31 in a mouse model of ischemic stroke (data not shown). Therefore, LM11A-31 could be relevant to HIV-1-infected individuals who are prone to other neuronal diseases, especially AD and stroke. LM11A-31 along with ART drugs could have a dual role in improving the outcome of comorbidities with HIV-1 and stroke or AD.

In conclusion, we report that LM11A-31 suppresses HIV-1 and has an additive effect on DRV. To some extent, it also decreases cytotoxicity and oxidative stress without causing autophagic dysregulation. More importantly, LM11A-31 alone and in combination with DRV decreases several proinflammatory cytokines and chemokine, resulting in a reduced inflammatory response in macrophages. Due to the inability of ART drugs to cross BBB and suppress HIV-1 in the brain, our present report has clinical relevance as LM11A-31 is hydrophilic and permeable to BBB. However, further studies are needed using an appropriate HIV-1 animal as well as dual (HIV-1+stroke) to fully realize the therapeutic potential of LM11A-31 in HIV-1 and other comorbid conditions.

## Data Availability

The original contributions presented in the study are included in the article/Supplementary Material, further inquiries can be directed to the corresponding author.

## Footnotes

1
https://clinicaltrials.gov/ct2/show/NCT03069014
